# Pointing in Visual Periphery: Is DF's Dorsal Stream Intact?

**DOI:** 10.1371/journal.pone.0091420

**Published:** 2014-03-13

**Authors:** Constanze Hesse, Keira Ball, Thomas Schenk

**Affiliations:** 1 School of Psychology, University of Aberdeen, Aberdeen, United Kingdom; 2 Department of Psychology, Durham University, Stockton-on-Tees, United Kingdom; 3 Neurology, University of Erlangen-Nürnberg, Erlangen, Germany; University of California, Merced, United States of America

## Abstract

Observations of the visual form agnosic patient DF have been highly influential in establishing the hypothesis that separate processing streams deal with vision for perception (ventral stream) and vision for action (dorsal stream). In this context, DF's preserved ability to perform visually-guided actions has been contrasted with the selective impairment of visuomotor performance in optic ataxia patients suffering from damage to dorsal stream areas. However, the recent finding that DF shows a thinning of the grey matter in the dorsal stream regions of both hemispheres in combination with the observation that her right-handed movements are impaired when they are performed in visual periphery has opened up the possibility that patient DF may potentially also be suffering from optic ataxia. If lesions to the posterior parietal cortex (dorsal stream) are bilateral, pointing and reaching deficits should be observed in both visual hemifields and for both hands when targets are viewed in visual periphery. Here, we tested DF's visuomotor performance when pointing with her left and her right hand toward targets presented in the left and the right visual field at three different visual eccentricities. Our results indicate that DF shows large and consistent impairments in all conditions. These findings imply that DF's dorsal stream atrophies are functionally relevant and hence challenge the idea that patient DF's seemingly normal visuomotor behaviour can be attributed to her intact dorsal stream. Instead, DF seems to be a patient who suffers from combined ventral and dorsal stream damage meaning that a new account is needed to explain why she shows such remarkably normal visuomotor behaviour in a number of tasks and conditions.

## Introduction

More than 20 years ago in a seminal paper, Milner et al. [1] described a severe case of visual-form agnosia (patient DF). Their observation that this patient (who suffers from bilateral damage to the ventral cortical stream) was unable to identify and recognise visually presented objects, but was able to use visual information to accurately control hand movements during reaching and grasping, contributed significantly to the development of a new model on how the brain processes visual information: the perception-action model [2]–[4]. In short, the model suggests that visual information is processed in different brain areas depending on the purpose for which the information is acquired: while visual information needed for the identification and recognition of objects is assumed to be primarily processed in ventral stream areas, visual information for the control of actions (such as reaching and grasping) is supposed to be primarily processed in dorsal stream areas of the brain.

Although the model has gained some support from behavioural and neuroimaging studies on neurologically intact humans, as well as physiological studies on monkeys over the last decades [4]–[6], behavioural studies on patient DF are still of crucial importance to sustain some of the key predictions of the model [7], [8]. Consequently, DF's perceptual and visuomotor performance has been tested extensively, resulting in more than 45 published studies comparing her perceptual and visuomotor performance in various tasks [8]. The observation that patient DF was consistently found to produce relatively accurate visuomotor behaviour even though her ventral pathways are extensively damaged has led to the conclusion that it is her intact dorsal stream that is responsible for her seemingly normal visuomotor performance. Furthermore, the finding that patients with dorsal stream damage (who suffer from optic ataxia) show the complementary pattern of deficits and retained functions with compromised visuomotor behaviour but intact perceptual performance [9]–[12] has strengthened the view that there is a double-dissociation in function between the dorsal and the ventral streams [2], [4].

However, recent imaging studies cast doubt on the presumption that DF only shows a circumscribed lesion to the ventral pathway. Already over a decade ago, James et al. [13] discovered, in a high-resolution anatomical MRI scan of DF's brain, that she suffered from an additional unilateral lesion to her left posterior parietal cortex (dorsal stream) and a general brain atrophy (enlarged sulci and ventricles). However, based on their functional data, they concluded that despite the small damage, these areas remained fully functional [13]. More recently, a detailed functional and structural analysis of DF's brain was provided by Bridge et al. [14]. According to their data, DF has significantly reduced cortical thickness in both hemispheres, in the lateral occipital cortex (LOC) as well as in the posterior intraparietal sulcus (IPS). Critically, the IPS is a dorsal stream structure that has frequently been associated with the occurrence of optic ataxia [3], [11], [15], [16]. Even though the data of Bridge et al. [14] indicates that DF's brain damage is considerably more widespread than originally assumed, it remains to date unclear whether the observed structural abnormalities beyond area LOC are actually functionally relevant. That is, we do not know whether DF shows any corresponding behavioural deficits.

As pointed out above, patients suffering from bilateral lesions to their posterior parietal cortex often show typical behavioural deficits. Specifically, they exhibit large reaching errors when they are asked to point to stimuli that are presented in their visual periphery while movements performed in central vision remain relatively normal [9]–[12], [17]. Moreover, if the lesions are bilateral, both visual hemifields and both hands should be affected by these errors to a similar extent [18]. In a previous study [19], we observed that DF shows impaired reaching and grasping behaviour when stimuli are presented in her visual periphery. However, in that study, we only examined her dominant right hand. Hence, we do not know whether her behavioural deficits are actually congruent with symptoms caused by bilateral damage to the dorsal stream. We originally suggested that DF's visuomotor deficits in visual periphery may be a secondary consequence of visual form agnosia making her movements more reliant on the availability of valid extra-retinal cues [19]. However, considering the recent imaging data of DF's brain [14], this interpretation may have been premature.

Surprisingly enough, most experiments have focussed exclusively on DF's right-hand performance leaving it unclear whether or not DF performs visuomotor tasks equally well with both hands. In order to find out whether DF's reaching deficits are compatible with a bilateral posterior parietal damage, we need to examine her pointing performance with her left and her right hands to peripheral targets presented in both visual fields. This is important as the question of whether DF's dorsal stream is functionally intact has significant implications for both the interpretation of previous reports on this patient and the validity of her position as key evidence for the perception-action model. If it turns out that DF's dorsal stream impairments are functionally relevant, one of the main interpretations of the perception-action model, namely that DF's preserved visuomotor competence (in central vision) is due to her intact dorsal stream, will be challenged.

Finally, studying DF's left hand performance in both free-viewing and fixation conditions is interesting for another reason. It has been suggested that the visuomotor mechanisms specialised for visuomotor control might be lateralised in the left hemisphere, thus providing a general right-hand advantage for (automatic) visuomotor tasks [20], [21]. The finding that grasping movements performed with the left hand are sensitive to size-contrast illusions (in both left and right handed participants) while movements performed with the right-hand seemed to be largely unaffected by visual illusions, has led to the hypothesis that left-handed actions might rely more strongly on perceptual (ventral) processing mechanisms [20]. If there is indeed a perceptual bias for movements performed with the left hand, we would expect that DF's left-handed visuomotor performance is generally worse than her right-handed performance in both free-viewing and fixation conditions. Specifically, we would predict that patient DF shows a larger difference (or dissociation) in accuracy between movements performed with the left and the right hand than neurologically healthy control subjects in all viewing conditions.

## Materials and Methods

### Participants

#### Patient DF

A patient with visual form agnosia (DF) participated in the experiment. Patient DF suffered a carbon monoxide intoxication in 1988 that led to extensive damage to her bilateral ventral lateral-occipital cortex, but left V1 and the fusiform gyrus largely intact [13]. DF's lesions correspond bilaterally with the location of the lateral occipital cortex (LOC) in the ventral stream of healthy subjects. Furthermore, James et al. [13] reported a small focus of damage in her left posterior parietal cortex posterior to the intraparietal sulcus. According to a more recent, detailed functional and structural analysis of DF's brain, the cortical thickness is significantly reduced in both hemispheres in the posterior intraparietal sulcus (IPS) as well as in area LOC [14].

Patient DF has trouble discriminating between different visual shapes, orientations, and distances, causing poor object recognition. Her luminance, colour, and texture perception is normal [1]. DF wore glasses correcting for a slight presbyopia. At the time of testing she was 58 years old.

Using static perimetry DF's left visual field was found to be normal. Her visual abilities in the upper right quadrant were also normal up to an eccentricity of 30°. There was an evident field loss in the inferior right visual field with 5°–10° of macular sparing (lower right quadranopia). Interestingly, DF showed a Riddoch phenomenon [22], i.e. her performance in the affected lower right quadrant improved with moving stimuli. In fact, she was able to detect moving stimuli in the right lower quadrant up to eccentricities of 25–30°, for more details, see: [19]. Further pre-tests performed in the lower right quadrant of her visual field revealed that she was able to discriminate between different colours and responded reliably to dots presented at 6.2° and 12.3° eccentricity but not to dots presented at 18.4° eccentricity (see data analysis section for more detail).

#### Control group

Nine female, right–handed and age-matched (mean age: 57 years, age range: 51–62 years) control participants were tested. All participants had normal or corrected to normal visual acuity and no history of neurological problems. All experiments were undertaken with the understanding and written consent of each participant in accordance with Durham University Review Ethics Board. Experiments were approved by the local ethics committee of the University of Durham (Department of Psychology) and in accordance with the principles of the Declaration of Helsinki. Control participants were paid £6 per hour.

### Apparatus and Stimuli

Pointing movements were measured using a 17” 3M MicroTouch Display (M1700SS) and fixation was controlled with an Eyelink II system (SR-Research). Movement onset was determined with a button box which was connected to the computer via a parallel port. The experiment was programmed in MATLAB using the Psychophysics Toolbox [23], [24] and the Eyelink Toolbox [25]. The pointing targets were red dots with a size of 1.3° of visual angle which were presented on a black background. The fixation cross was white with a size of 1° of visual angle.

### Procedure

Participants sat on a height-adjustable chair in a well-lit room. The touch screen monitor was placed centrally in front of them on the table with a viewing distance of 50 cm. A chinrest was used to maintain a stable head position with a constant viewing distance throughout the experiment. Between the chinrest and the monitor, a button box was placed on the table (vertically aligned to the participants' midline). The distance between the start button and the centre of the screen was 35 cm.

At the beginning of each trial participants pressed a start button with the index finger of their responding hand. Each trial started with the presentation of a fixation cross in the centre of the screen. After a fixation period of 1 s the target dot was presented. There was a preview period of 1 s after which an auditory go-signal (100 ms) signalled to participants to point to the red target dot presented on the screen. As soon as participants lifted their finger from the start-button the target disappeared (open-loop condition). An open-loop viewing condition was chosen as we have previously shown that DF's performance in closed- and open-loop conditions is comparable for both fixation and free-viewing tasks [19]. It should also be noted that previous investigators could demonstrate the typical pattern of ataxic deficits (pronounced errors in the contralesional visual field for movements performed with the contralesional hand) in a patient with predominantly unilateral dorsal-stream damage while using an open-loop paradigm [26]. As the targets were visible until the finger was lifted off the start position (during the reaction time interval), movements are still programmed based on the real-time computations of the dorsal stream according to the perception-action model [5]. Participants were instructed to point as accurately as possible and to move at a natural speed. Previous studies investigating optic ataxia have revealed consistent hand and field effects in both speeded [11], [18] and unspeeded tasks [12], [17], [27]. As clinical examinations usually require natural, and thus unspeeded movements from the patients, and as we have used the same paradigm with DF previously [19], we kept this instruction for consistency.

The experiment consisted of two different viewing conditions which were completed in separate blocks: the free-viewing block always preceded the fixation block meaning that all control participants completed the experiment using the same order of blocks as patient DF. In the free viewing condition, the fixation cross was extinguished at the moment the target dot appeared on the screen. Thus, the fixation cross was not present during both the pre-view period and during the pointing movement, meaning that participants could move their eyes freely. In the fixation condition, the fixation cross remained visible throughout, and participants were instructed to keep fixation at the cross until they had finished their pointing movement toward the target.

**Figure 1:**
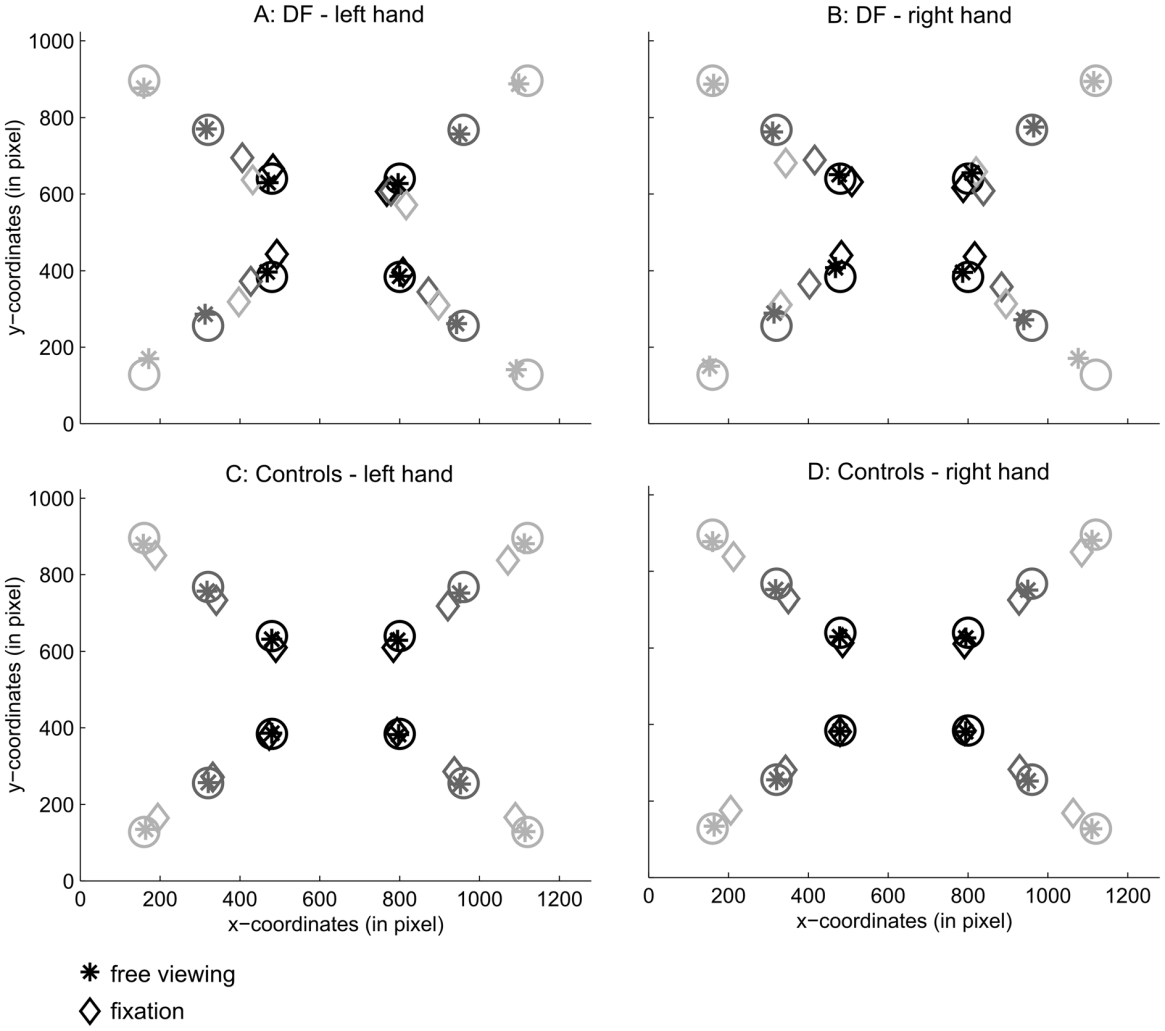
Average pointing position of patient DF and the control participants for all target positions. Movements are depicted separately for the left hand (left panel) and right hand (right panel) for patient DF (top row) and the control participants (bottom row). Pointing movements to the different visual eccentricities are colour-coded: black for near targets (6.2° of visual angle), dark grey for targets presented at mid eccentricity (12.3° of visual angle) and light grey for targets presented at far eccentricities (18.4° of visual angle). The position of the target is indicated by a circle, pointing movements performed in the free viewing conditions are indicated by an asterisk, and pointing positions in the fixation condition are represented by a diamond. Note that patient DF frequently missed targets presented at the furthest eccentricity (18.4°) in the inferior right visual field in the fixation condition. We included all trials (N = 4) in which DF perceived the target and performed a pointing movement (see methods section for details).

The target was presented at 12 different positions on the screen (Figure 1, grey circles). Targets were presented in all four quadrants of the monitor at three different eccentricities along the 45° diagonal of each quadrant. Near-distance targets were presented at 6.2° of visual eccentricity, mid-distance targets at 12.3° of visual eccentricity, and far-distance targets at 18.4° of visual eccentricity. Target positions were presented randomly throughout the experiment, and each target was presented 8 times resulting in a total of 96 trials per block. Both blocks (fixation and free-viewing) were performed with the right and the left hand (total of 384 trials). All participants performed the tasks with the right hand first (the same order as DF). Apart from two control participants, all participants (including patient DF) completed the tasks on two different days. Two of the control participants had an extended break between performing the experiment with their right and their left hands.

Before the start of the experiment the Eyelink system was adjusted and calibrated. A recalibration of the Eyelink system was implemented after each block and participants performed 8 practice trials to familiarise themselves with the task. During the experiment a drift correction was applied every ten trials.

### Data Analysis

In all trials the eye-movements of participants were monitored on-line by the experimenter (CH and KB). When participants failed to keep fixation during the pointing movement, or started the movement before the go-signal, the trial was repeated later in the block (at a random position). On average, control participants lost fixation in 26 out of 192 (13.5%) pointing trials that required fixation. DF showed a comparably good fixation performance: 13 pointing trials were repeated (7%).

Pointing errors were computed as absolute error and variable error. The absolute error was defined as the absolute distance (in mm) in 2D between the finger end-position at the moment a touch on the monitor was registered and the centre of the target dot. The variable error (end point variability) was computed using the following formula: Variable Error  =  Square Root ([SD(d_x_)]^2^+ [SD(d_y_)]^2^) with SD being the standard deviation, and d_x_ and d_y_ being the differences in the coordinates of the target centre and the final pointing position [28]. Standard deviations can be used as an appropriate measure of dispersion since it has been shown that the distribution of endpoints in unconstrained pointing movements (such as in the current study) tends to be normally distributed [29].

The data was averaged across target positions presented at the same eccentricities within one visual field, that is, we averaged across presentations in the upper and the lower visual fields. This meant that for each visual field (left and right) we obtained an average pointing error for targets presented at near, mid, and far distance from fixation (16 trials per condition). As DF had trouble perceiving targets presented at the far eccentricity (18.4°) in the lower right visual field, she missed some of the targets presented at this location. Her data for this condition is therefore based on all trials in which she performed a pointing movement (12 trials for the left and the right hand respectively). Pointing accuracy was similar for targets presented in the lower and the upper visual field (cf. Figure 1). All pointing data can be made available on request.

For all statistical comparisons we used modified t-tests specifically developed for single-case studies [30]. When multiple tests were computed we adjusted the p-values using a Bonferroni correction.

## Results

### Free viewing

In order to test whether participants looked in the direction of the pointing target in the free viewing conditions, we calculated the absolute distance (in 2D) of the fixation location from the centre of the pointing target at the moment the start-button was released. Patient DF's average fixation location was about 1.5° of visual angle from the centre of the near-distance targets, about 2.0° of visual angle from the centre of the mid-distance targets, and about 3.5° of visual angle from the centre of the far-distance targets. Overall, DF fixated similarly closely to the targets at all eccentricities as the control participants (all p>.32). The average distance between target centre and fixation location for the control participants was 1.3°, 1.8° and 2.9° of visual angle for the close, mid and far-distance targets respectively. This data shows that in the free viewing condition participants tended to fixate closely to the location of the pointing target.

#### Distance error

**Figure 2:**
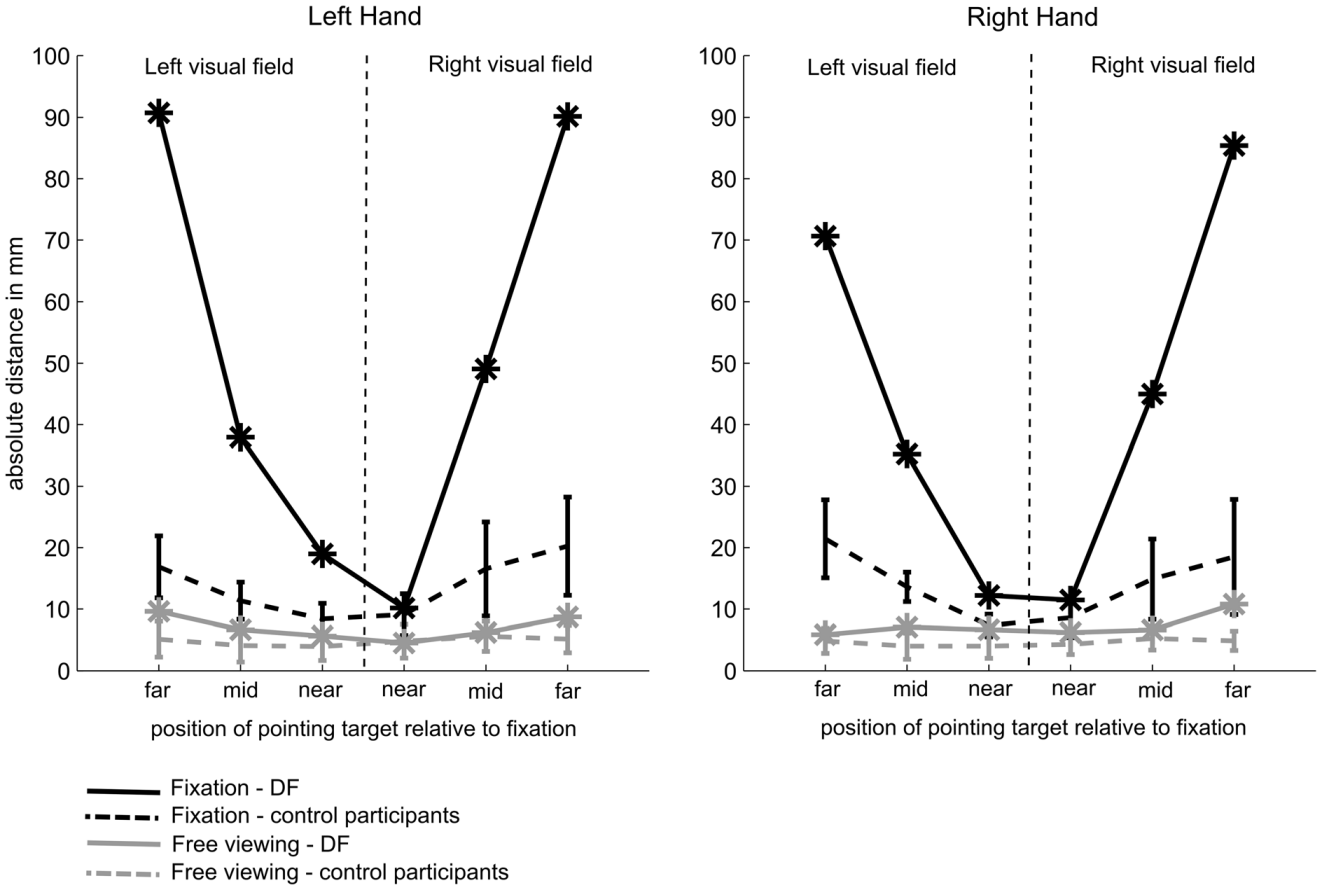
Average 2D-pointing-distance from the target centre as a function of target eccentricity and visual field. Pointing errors are depicted separately for pointing movements performed with the left hand (left panel) and movements performed with the right hand (right panel). Grey lines represent movements executed in the free-viewing conditions and black lines represent movements executed in the fixation conditions. The performance of the controls is represented as dashed lines and DF's performance as solid lines. Error bars depict the sample standard deviation.

Figure 2 shows that DF pointed relatively accurately to all targets in the free-viewing conditions. In order to statistically test whether DF's pointing accuracy differed from the pointing accuracy of the control participants, we calculated modified t-tests for single case statistics [30]. Results of these tests revealed that DF was as accurate as the controls in all conditions which required pointing with the left hand (all p>.17). Similarly, when pointing with her right hand she was as accurate as the controls in all but one condition. The only condition in which we observed a significant difference was when DF had to point to targets presented at 18.4° in the right visual field, t(9) = 3.516, p = .007 (for all other conditions p>.16). This finding is likely to be related to the fact that DF has problems in perceiving targets presented at this eccentricity in the lower right visual field. Overall, the results confirm that in free-viewing conditions, DF performs largely normally (within the range of the healthy control participants) with both her right hand and her left hand.

#### Variable error

**Figure 3:**
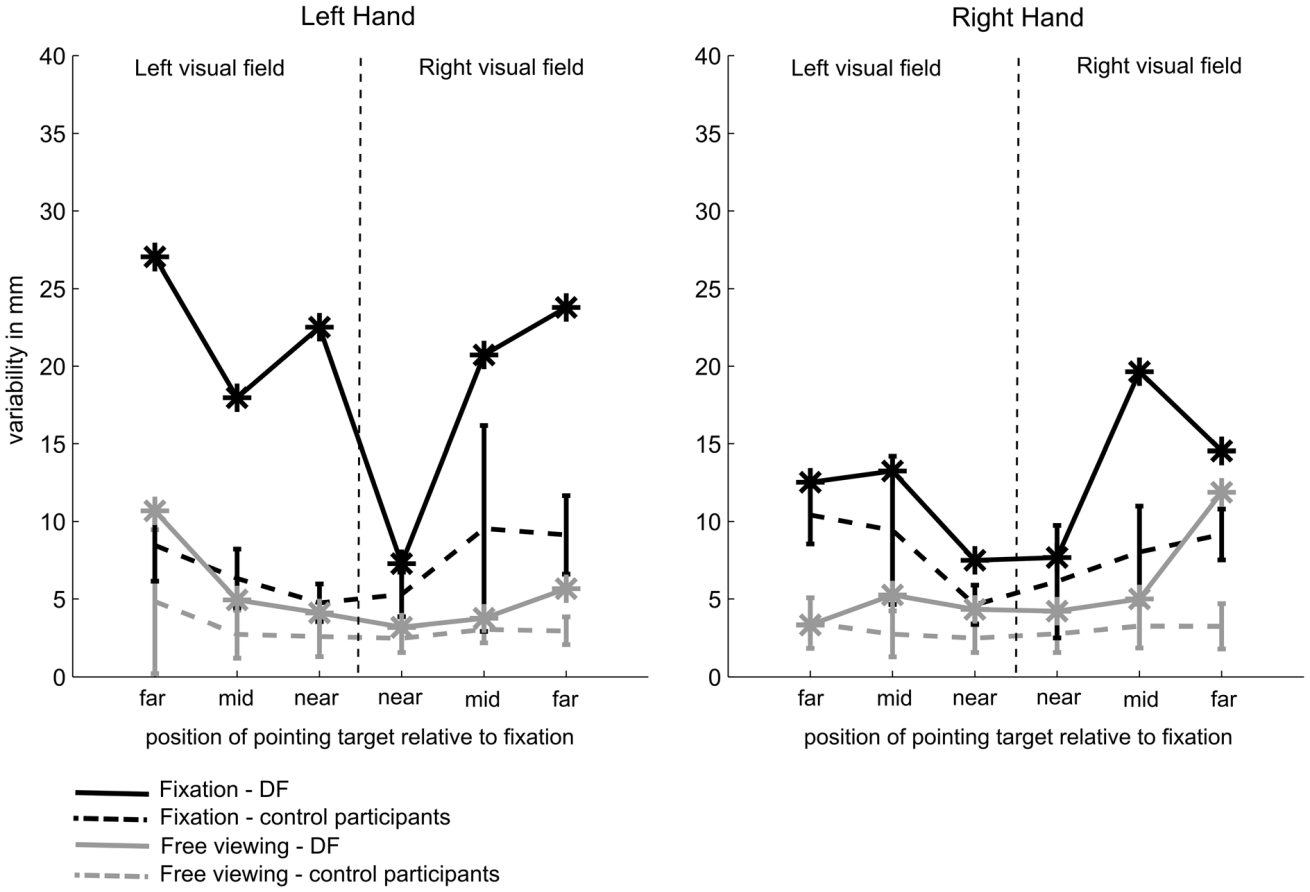
Average 2D-pointing-variability as a function of target eccentricity and visual field. Pointing variability is depicted separately for pointing movements performed with the left hand (left panel) and movements performed with the right hand (right panel). Grey lines represent movements executed in the free-viewing conditions and black lines represent movements executed in the fixation conditions. The performance of the control participants is represented as dashed lines and DF's performance as solid lines. Error bars depict the sample standard deviation.

Figure 3 shows the variable error for DF and the control participant for pointing movements performed with the left and the right hand in the free viewing conditions (grey lines). Even though numerically DF's pointing movements seem to be slightly more variable than those of the controls, modified t-tests confirmed that DF showed similar variability as the controls in all but one condition. Similarly as for the distance error, DF showed an increased variability compared to the controls when she had to point with her right hand to targets presented in the right visual field at an eccentricity of 18.4° of visual angle, t(9) = 5.65. p = .0005.

### Fixation

#### Distance error

When the pointing targets were presented in visual periphery and DF was asked to keep fixation during movement execution, her performance decreased drastically. As shown, in Figures 1 and 2, she underestimated the eccentricity of the target dots considerably, resulting in increased pointing errors for increasing visual eccentricities. Using the modified t-test [30] and a Bonferroni adjusted significance level of p = .0083 (six tests for pointing movements performed with each hand), we confirmed that DF's pointing performance was significantly worse than the performance of the controls for all targets presented at both the mid (12.3° of visual angle) and far visual eccentricities (18.4° of visual angle) in both the left and right visual field and independent of whether pointing movements were executed with the left or the right hand (all one-tailed p<.001). For targets presented relatively close to fixation (6.2° of visual angle) DF was as good as the control participants when pointing toward targets presented in the right visual field with both her left hand, t(8) = 0.30, p = .39, and her right hand, t(8) = 0.86,p = .20. Furthermore, at near eccentricities (6.2° of visual angle) she was marginally worse than the controls when pointing with her right hand in the left visual field, t(8) = 2.53, p = .018, and she showed a significant impairment for pointing movements performed with her left hand in the left visual field, t(8) = 4.01, p = .002. This analysis shows that, independent of which hand was used and in which side of the visual field targets were presented, DF's performance was significantly impaired when movements had to be performed to targets presented at mid and far eccentricities.

To test whether DF's performance was modulated by the side of presentation (i.e. visual target presented in the right versus left visual field) and/or by the effector that is used for the movement (i.e. right versus left hand), we conducted a 2 (visual field)×2 (hand)×3 (eccentricity) repeated-measures ANOVA on all trials performed by DF. Please note that DF missed 4 out of the 8 targets presented in the lower right visual field when pointing with the left as well as when pointing with the right hand. Hence, the ANOVA is based on a total of N = 12 trials per condition (i.e. 144 trials in total). As expected, this analysis revealed a significant main effect of eccentricity, F(2,22) = 281.14, p<.0001. There were, however, no main effects of hand (p = .33) and visual field (p = .45) on the absolute pointing error. The interaction effect between eccentricity and visual field was marginally significant, F(2,22) = 3.67, p = .05, indicating that the effect of target eccentricity was slightly larger on movements performed to the right visual field. All other interaction effects were not significant (p>.30).

Finally, we averaged the data over all visual eccentricities and conducted difference tests for single subject data [31]. This allowed us to test whether the factors visual hemifield (left versus right) or hand (left versus right) affected DF's pointing performance more than that of control subjects.

Hand effect: As expected, the statistical analysis confirmed that DF's pointing performance was significantly worse compared to the performance of the controls for both when pointing with the left hand, t(8) = 7.20, p<.0001, and when pointing with the right hand, t(8) = 6.5, p<.0001. However, the difference test revealed no stronger discrepancy between the performance of the two hands for DF than for the controls, t(8) = 1.84, p = .10, confirming that DF's performance was similarly impaired for both hands.

Visual field effect: The statistical test comparing DF's average pointing performance in the left and the right visual field with the controls confirmed a significant impairment in both visual fields (left VF: t(8) = 10.30, p<.0001; right VF: t(8) = 4.46, p = .001). Again the difference test gave no indication that the discrepancy between the performance in the two visual fields was greater for DF than for the controls, t(8) = 1.07, p = .32, confirming that DF's performance was impaired to a similar extent in both visual fields.

#### Variable Error

The variable error of DF and the control participants in the fixation conditions is depicted in Figure 3. Visual inspection suggests that even though DF seems to show slightly increased variable error in the fixation conditions relative to the control participants, the pattern seems to be less consistent than for the absolute pointing error. Modified t-tests [30] with a Bonferroni adjusted significance level of p = .0083 (six tests for pointing movements performed with each hand) confirmed that DF's pointing performance was significantly more variable than the performance of the controls at all eccentricities for movements performed with the left hand in the left visual field (all p<.0004). In contrast, when pointing movements with the left hand into the right visual field were required, DF's pointing variability was similar to those of the controls at both 6.2° of visual eccentricity, t(9) = 1.30, p = .22, and 12.3° of visual eccentricity, t(8) = 1.6, p = .15. At far eccentricities (18.4° of visual angle), DF was significantly more variable than the control participants, t(8) = 5.49, p = .0006. In contrast, when pointing with the right hand in the left visual field, DF's variability was similar to the control participants across all eccentricities (all p>.06). For pointing movements executed with the right hand in the right visual field, we observed a significantly higher variable error for DF only for movements performed to the targets presented at 12.6° of visual angle, t(8) = 3.71, p = .006. Thus, DF showed a similar variability as the control participants when pointing with her right hand for all but one target position.

## Discussion

The case of patient DF has fundamentally shaped our view on how visual information is processed for perception and action in the human brain [3], [4]. Since the first detailed description of her case by Milner et al. [1], DF has been portrayed as the ideal case to test the predictions of the perception action model [7], [32]. As she is considered a patient with selective and circumscribed ventral-stream damage, it is argued that her near-to-normal visuomotor behaviour confirms that an intact dorsal stream is sufficient to generate normal visually guided behaviour.

When it was first discovered ten years ago that DF had a small additional lesion in the posterior parietal cortex, this damage was dismissed as functionally irrelevant [13]. However, very recently, we observed that patient DF shows behavioural impairments when performing actions in visual periphery [19] that are similar to those observed in patients with optic ataxia (dorsal stream damage). This observation, together with the publication of a new functional and structural report of DF's brain revealing a bilateral thinning of the grey matter in the posterior portions of the IPS [14] has given rise to the speculation that DF may be also suffering from optic ataxia. In our current study, we systematically investigated DF's pointing behaviour in visual periphery when executing movements with the left and the right hand in both visual fields. We found that DF shows large and relatively symmetrical pointing errors in both visual fields that occur independently of the hand with which they are performed. Therefore, DF shows the same visuomotor problems that are typically observed in patients with optic ataxia who suffer from bilateral damage to the dorsal stream areas. Hence, our findings suggest that DF's bilateral dorsal stream atrophies should not prematurely be dismissed as functionally irrelevant. In fact, our data provides the first tentative evidence that DF's dorsal stream functions may be partly compromised as well. Clearly, we cannot completely rule out the possibility that DF's problems in pointing to peripheral targets are the result of her ventral stream damage. However, the fact that this type of pointing deficit has so far only been described after damage involving the dorsal streams [17], suggests to us that DF's bilateral abnormalities in her dorsal stream are the most likely cause of her pointing deficits.

If it can be confirmed in further studies that DF's dorsal stream damage is behaviourally relevant, this finding will have far reaching implications for the perception-action model; for example, it could no longer be assumed that DF's preserved visuomotor capabilities in central vision can be attributed to her intact dorsal stream. Instead, the case of DF would provide evidence that the visuomotor system works quite efficiently even when there is damage to both dorsal and ventral stream areas.

How else can we then explain DF's surprisingly good visuomotor behaviour, if we assume that it is not based on processing in the intact dorsal stream? Schenk [33] argued that DF's visuomotor robustness is the result of the intrinsic sensory redundancy of human sensorimotor control. That is, success in most sensorimotor tasks does not rely on one single sensory cue but is based on a combination of different cues. Selective loss of some of those cues will therefore not destroy performance, but will instead make the patient's visuomotor behaviour less flexible and more dependent on cues which are still usable. In support of this idea, several researchers have demonstrated that DF is more affected by changes to the array of available sensory cues in her sensorimotor behaviour than healthy controls [34], [35]. How can we explain in this context the observation that DF can only accurately point to targets when they are presented in central vision? One possibility is that DF uses gaze direction to guide her pointing movements. This extra-retinal cue only provides useful information about the position of the target when eye and hand movements are aligned, but not when participants are instructed to fixate at a different position in space away from the pointing target (as is the case for targets presented in visual periphery). Support for the account that gaze-position is an important cue in pointing is provided by studies that show reliable and consistent effects of gaze direction on pointing movements even in neurologically intact participants [36]–[39].

Proponents of the perception-action model might counter that DF's dorsal stream damage may be severe enough to interfere with pointing movements performed to peripheral targets but at the same time so restricted that other visuomotor behaviour can still be supported by the dorsal stream (such as pointing in free-viewing conditions). In response to this, we can only point out that DF's visuomotor deficits are in fact no less pronounced than the deficits of most other patients with optic ataxia whose brain damage has been properly documented [9], [11]. Furthermore, this view is also problematic as it was argued by Rossetti et al. [9] that the visuomotor function of the dorsal stream may actually be restricted to the processing of visual targets presented in periphery. Thus, proponents of the perception-action model seem to be left with an unattractive choice: They either accept that DF - their best example of a patient with pure ventral stream damage - has in fact also damage to her dorsal stream; or they withdraw their claim that selective optic ataxia provides evidence for selective dorsal stream damage [2], [3]. In the latter case, the only compelling neuropsychological disorder demonstrating the presumed relevance of the dorsal stream for visuomotor control would be dismissed.

Finally, it is worth commenting on our observation that, in the free viewing conditions, DF's left hand performance is as good as that of the control participants. Previous studies suggested that the visuomotor mechanisms mediating the visuomotor control of target-directed movements have evolved preferentially in the left hemisphere, thus providing an overall right-hand advantage for visuomotor tasks such as reaching and grasping [20], [21]. Specifically, the finding that left-handed movements (but not right-handed movements) are sensitive to visual illusions led to the suggestion that the left hand may depend more strongly on ventral stream processing [20].

In line with this argument, we observed that when DF performed pointing movements in visual periphery, she showed increased pointing variability when pointing with her left hand. In contrast, movements performed with the right hand were similar in variability to those performed by the control participants in the fixation conditions. Hence, even though DF's average pointing error (distance error) was similar for the left and the right hand in the fixation conditions, the increased variability for left-handed pointing might indeed indicate increased ventral stream involvement in the planning and execution of these movements. This interpretation is, however, in conflict with the data obtained from the free-viewing condition. The free-viewing condition offers the opportunity to disentangle the effects of dorsal-stream damage from the putative effects of ventral-stream damage. Typically, dorsal stream damage leading to optic ataxia will not affect movements performed under free-viewing conditions. In contrast to this, ventral stream damage is assumed to affect left-hand behaviour in free-viewing conditions. Following this logic, the free-viewing condition provides a good test of the effects of ventral stream damage on pointing behaviour. That is, if it were true that the ventral stream is specifically involved in the control of left-hand but not right-hand behaviour, a clear left-hand inferiority should be observed in patient DF in the free-viewing conditions. However, our data did not confirm this prediction as DF was able to perform left-handed movements as accurately as control participants when free-viewing was allowed.

## Conclusion

In this study we tested whether DF's reported dorsal stream damage causes any corresponding behavioural deficits. Our results indicate that when performing pointing movements in visual periphery, DF is consistently impaired regardless of whether the stimuli are presented to her right or left visual hemifield and regardless of whether she is using her right or left hand for pointing. Such a pattern of errors is usually interpreted as a clear sign of bilateral optic ataxia. Our data therefore suggests that DF's dorsal stream functions are bilaterally impaired. This finding is difficult to reconcile with the explanation for DF's good visuomotor performance provided by the perception-action model. According to the assumptions of the perception-action model, DF's intact dorsal streams are the source of her normal visuomotor behavior in central vision. Our findings, however, suggest that DF is capable of good visuomotor behaviour (at least in some tasks under some conditions) despite damage to both her ventral and dorsal streams. Hence, we conclude that neither ventral nor dorsal streams are critical for all aspects of visually-guided behavior but that the visuomotor system receives sensory contributions from a great range of brain structures.
